# Dual-Specificity Phosphatase 11 Is a Prognostic Biomarker of Intrahepatic Cholangiocarcinoma

**DOI:** 10.3389/fonc.2021.757498

**Published:** 2021-09-29

**Authors:** Lin Xu, Peng Wang, Wei Zhang, Weiran Li, Tao Liu, Xu Che

**Affiliations:** ^1^ Department of Hepatobiliary and Pancreatic Surgery, National Cancer Center/National Clinical Research Center for Cancer/Cancer Hospital & Shenzhen Hospital, Chinese Academy of Medical Sciences and Peking Union Medical College, Shenzhen, China; ^2^ Department of Pancreatic and Gastric Surgery, National Cancer Center/National Clinical Research Center for Cancer/Cancer Hospital, Chinese Academy of Medical Sciences and Peking Union Medical College, Beijing, China; ^3^ Department of Oncology Rehabilitation, Shenzhen Luohu People’s Hospital, Shenzhen, China

**Keywords:** DUSP11, prognosis, biomarker, intrahepatic cholangiocarcinoma, extrahepatic cholangiocarcinoma

## Abstract

**Background:**

Cholangiocarcinoma (CCA), including intrahepatic (iCCA), perihilar (pCCA), and distal (dCCA) CCA, is a highly aggressive malignancy originating from bile duct. The prognosis of CCA is very poor, and the biomarker study is unsatisfactory compared with other common cancers.

**Materials and methods:**

In our study, we investigated the expression of dual-specificity phosphatase 11(DUSP11) in eight pairs of iCCAs, pCCAs, and dCCAs, and their corresponding tumor-adjacent tissues, as well as their tumor-adjacent tissues with qPCR. Moreover, we investigated the expression of DUSP11 in 174 cases of CCAs with immunohistochemistry, including 74 iCCAs, 64 pCCAs, and 36 dCCAs. We classified these patients into subsets with low and high expressions of DUSP11, and evaluated the correlations between the DUSP11 subsets and clinicopathological factors. With univariate and multivariate analyses, we assessed the correlation between DUSP11 and the overall survival (OS) rates in these CCA patients.

**Results:**

In all the CCA subtypes, DUSP11 was elevated in CCAs compared with their paired adjacent tissues. In iCCA, pCCA, and dCCA, the percentages of DUSP11 high expression were 44.59%, 53.85%, and 55.56%, respectively. In iCCA, high DUSP11 expression was significantly associated with an advanced T stage and a poor prognosis. However, the prognostic value of DUSP11 in pCCA and dCCA was not significant. To decrease the statistical error caused by the small sample size of the dCCA cohort, we merged pCCA and dCCA into extracellular CCA (eCCA). In the 101 cases of eCCA, DUSP11 expression was also not significantly associated with the prognosis.

**Conclusions:**

DUSP11 expression was associated with tumor infiltration and the OS rate in iCCA, but not in pCCA and dCCA. DUSP11 was an independent biomarker of iCCA indicating a poor prognosis. Our results suggested that a high expression of DUSP11 was a post-operational risk factor, and detecting DUSP11 could guide the individual treatment for patients with CCA.

## Introduction

Cholangiocarcinoma (CCA) is a highly aggressive malignancy with extremely poor prognoses. Anatomically, CCA is further classified as three subtypes including intrahepatic (iCCA), perihilar (pCCA), and distal (dCCA) CCA based on their origin in the biliary tree (1). pCCA is the most prevalent type of CCA accounting for about 50%–60% of the total CCA cases, while dCCA and iCCA accounted for 20%–30% and 10%, respectively (2). The motility and morbidity of CCA are increasing rapidly in the recent decades, but the treatment options have few progresses (3). China has the highest incidence of CCA worldwide, and whether Chinese CCA patients have special molecular features is still unknown. In general, the rate of radical surgical resection of CCA is quite low, and the treatment options of unresectable or advanced-stage CCA are an urgent need. CCAs usually have poor responses to the classical adjuvant therapies such as chemotherapy or radiotherapy. Till 2020, there emerged the first FDA-approved target drug of CCA, pemigtinib, which was used for CCA with FGFR2 fusion or mutation (4). Overall, the 5-year overall survival rate (OS) of CCA is very unsatisfactory, remaining approximately 30% after radical resection (5). More biomarkers of CCA should be investigated because biomarker discovery is the initiation to find new drug targets and new treatment therapy.

Protein phosphorylation is a key post-translational modification mainly regulated by serine/threonine phosphatase and tyrosine phosphatase. Protein tyrosine phosphatase (PTP) family participates in numerous processes such as signal transduction and cell proliferation, *via* dephosphorylating the phosphor-tyrosine of substrates (6). In humans, PTPs consist of 107 members and are divided into four main subgroups, which are type-I cysteine-based PTP (including classical PTPs and dual-specific phosphatase), type-II cysteine-based PTP, type-III cysteine-based phosphatases, and aspartic acid-based PTPs (7). These PTPs have different tissue specificities, substrates, and functions. Genetic and epigenetic alterations in the PTP genes can result in aberrant tyrosine phosphorylation, and consequently lead to diverse effects including an uncontrollable cell proliferation and tumorigenesis (8, 9). Interestingly, both the tumor suppressing role and oncogenic functions of PTPs have been showed in cancer, and the putative oncogenic or tumor suppressive functions of PTP are considered to rely on the cellular context.

Dual-specificity phosphatases (DUSPs) have a dephos-phorylating activity to both threonine/serine and tyrosine residues (10). There are 61 DUSPs out of the 107 PTP members, which have heterogeneous forms and functions, and are further classified based on the specific domains and sequence similarity. DUSP11, also known as PIR1 (phosphatase interacting with RNA and ribonucleoprotein 1), is a unique member of atypical DUSPs which could bind directly to RNA and possess RNA 5’-triphosphatase and diphosphatase activities (11, 12). DUSP11 converts the 5’ triphosphate of microRNA precursors to a 5’ monophosphate, and regulates cellular noncoding RNAs levels (12–14). In addition to a catalysis towards RNA, more evidence showed that DUSP11 could also dephosphorylate proteins. For example, DUSP11 could attenuate lipopolysaccharide-induced macrophage activation by targeting TGF-β-activated kinase 1 (15). DUSP11 was considered to participate in cancer progression by several previous studies (16, 17), but its functions and regulation mechanisms in cancer are still unclear to date. The tissue specificity and functions in tumor of DUSP11 have not been well studied.

In our study, we investigated the expression of DUSP11 in 174 cases of CCAs, including 74 iCCAs, 64 pCCAs, and 36 dCCAs. Moreover, we classified the patients into subsets with low and high expressions of DUSP11, and evaluated the clinicopathological factors in these subsets. With univariate and multivariate analyses, we assessed the correlation between DUSP11 and the OS rate in 74 iCCAs, 64 pCCAs, and 36 dCCAs.

## Materials And Methods

### Patients and Ethics

A total of 258 patients were diagnosed with CCA in Chinese Academy of Medical Sciences and Peking Union Medical College and National Cancer Center Shenzhen Hospital from 2009 to 2016, which formed the primary cohort. A total of 174 cases of CCAs, including 74 iCCAs, 64 pCCAs, and 36 dCCAs, were selected from the primary cohort into the validation cohort, if they followed the criteria: (1) radical surgery with clear surgical margin was performed; (2) available formalin-fixed tumor tissues for IHC; (3) available follow-ups more than 3 months and complete medical records; and (4) no history of other malignancies. All samples were obtained with a prior consent from patients. The study was approved and supervised by the Ethics Committee of Chinese Academy of Medical Sciences and Peking Union Medical College and National Cancer Center Shenzhen Hospital.

### Quantitative Real-Time PCR Analysis

A total of eight consecutive iCCAs, pCCAs, and dCCAs, and their corresponding tumor-adjacent tissues were collected for qPCR. Total mRNA was extracted from the frozen tissues using the TRIzol reagent (Thermo Fisher Scientific, Waltham, MA, USA), and then converted into cDNA using the ReverTra Ace qPCR RT kit (TOYOBO, Japan). Quantitative real-time PCR was performed using the SYBR Green Master (Roche, USA) and Light Cycler Roche 480 PCR instrument. The mRNA level was standardized with the 2^-ΔΔ^Ct method by normalization to GAPDH. The primer sequences were as follows:

DUSP11, forward:5’-GGCTGCCGAGTCTTTTCCT-3’,Reverse5’-TTTCCACCTTTCGGGGATGTG-3’; GAPDH, forward:5’-GGAGCGAGATCCCTCCAAAAT-3’, reverse: 5’-GGCTGTTGTCATACTTCTCATGG-3’.

### Immunohistochemistry

Immunohistochemistry was performed with a streptavidin peroxidase complex method. Briefly, the paraffin-embedded tissues were deparaffinized and rehydrated with xylene and graded alcohol. To inactivate the endogenous peroxidase, 3% hydrogen peroxide was used, and then, the slides were incubated in a citrate buffer (pH = 6.0) for the optimal antigen retrieval. The unspecific binding was blocked by incubation in 1% bovine serum albumin for 30 minutes. Primary antibody of DUSP11 (Santa Cruz Biotechnology, catalog: sc-393220) was used to incubate the tissues at 4°C overnight. Phosphate buffered saline was used to rinse the slides three times, and secondary antibodies labeled with streptavidin-biotin-peroxidase reagent were used to incubate the tissues for 1 hour. After that, slides were treated with the 3,3’-diaminobenzidine solution for 10 minutes for visualization. Slides were counterstained with hematoxylin and mounted at last.

### Immunohistochemistry Results Evaluation

The IHC results were semi-quantified by two independent pathologists who were unaware of the clinical data. The final IHC scores was evaluated as the scores of the percentage of positive-stained cells multiplied by the scores of staining intensity. In brief, the scores of staining intensity were defined as: score 0 for negative staining, score 1 for weak staining, score 2 for moderate staining, and score 3 for strong staining. The scores for positive-stained cells were set as follows: score 1 for <25% of positive cells; score 2 for 25%–50% of positive cells; score 3 for 50%–75% of positive cells; and score 4 for 75%–100% of positive cells. The final IHC score ranged from from score 0 to 12, and was divided into subsets with different DUSP11 expression according to the cut-off, which was defined by the receiver operating characteristic (ROC) curve.

### Statistical Analysis

SPSS 25.0 (IBM, Chicago, IL, USA) and GraphPad prism 5.0 software (California Resources Corporation, Los Angeles,CA, USA) were used for statistical analyses. The chi-square test was used to analyze the correlations between DUSP11 and the clinicopathological factors. The univariate analysis was analyzed with the log-rank test, and the survival curves were plotted with the Kaplan-Meier method. The Cox proportional hazards regression model was applied to identify the independent prognostic factors. P-values less than 0.05 in all experiments were considered statistically significant.

## Results

### Expression of Dual-Specificity Phosphatase 11 in Cholangiocarcinoma Tissues and Tumor-Adjacent Tissues

**Figure 1:**
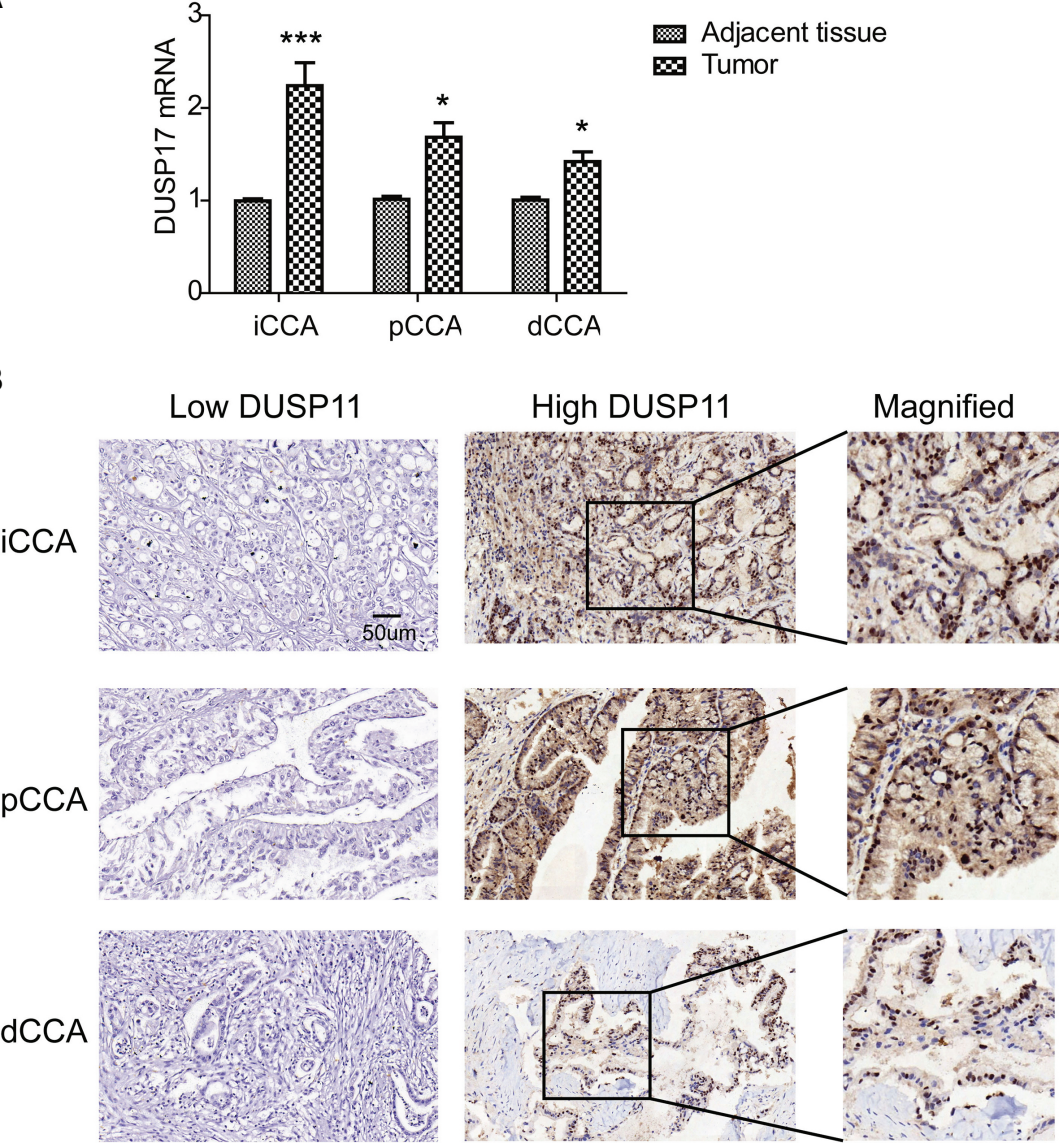
Expression of DUSP11 in CCA tissues. **(A)** The expression of DUSP11 was detected with qPCR in eight pairs of iCCAs, pCCAs, and dCCAs, as well as their corresponding tumor-adjacent tissues. * represents *P *< 0.05 and *** represents *P *< 0.0001, calculated with paired t-test. **(B)** DUSP11 expression was investigated in 174 cases of CCAs, including 74 iCCAs, 64 pCCAs, and 36 dCCAs, by IHC. Representative images of low and high DUSP11 expressions were displayed.

The expression of DUSP11 was detected with qPCR in eight pairs of iCCAs, pCCAs, and dCCAs, as well as their corresponding tumor-adjacent tissues (**Figure 1A**). In these tissues, DUSP11 expressions in iCCAs, pCCAs, and dCCAs were substantially higher than those in their paired adjacent tissues. To better depict the expression of DUSP11 in CCA, the DUSP11 expression was investigated by IHC in 174 cases of CCAs, including 74 iCCAs, 64 pCCAs, and 36 dCCAs. In consistent with the DUSP11 function as a phosphatase toward phosho-RNA, the intracellular localization of DUSP11 was in the cell nucleus in CCA (**Figure 1B**). In iCCA, pCCA, and dCCA, the percentages of DUSP11 high expression were 44.59%, 53.85%, and 55.56%, respectively (**Table 1**). The basic information of CCA patients is shown in **Table 1**, including the sex, age, tumor size, differentiation, and T/N/M/TNM stage of the patients. The results of basic patients characters were consistent with previous studies (5, 18), supporting the validation of our cohort.

**Table 1:** Basic information of CCA patients.

Clinicopathologic parameters		iCCA		pCCA		dCCA	
		n	percentage	n	percentage	n	percentage
Age (years)	<60	45	60.81%	40	61.54%	27	75.00%
	≥60	29	39.19%	25	38.46%	9	25.00%
Sex	Male	37	50.00%	46	70.77%	21	58.33%
	Female	37	50.00%	19	29.23%	15	41.67%
Tumor size^#^ (cm)	<3/5 cm	34	45.95%	37	56.92%	26	72.22%
	≥3/5 cm	40	54.05%	28	43.08%	10	27.78%
Differentiation	Well	17	22.97%	31	47.69%	17	47.22%
	Moderately	36	48.65%	25	38.46%	12	33.33%
	Poorly	21	28.38%	9	13.85%	7	19.44%
T stage	T1 + 2	52	70.27%	28	43.08%	16	44.44%
	T3 + 4	22	29.73%	37	56.92%	20	55.56%
N stage	N0	49	66.22%	52	80.00%	24	66.67%
	N1	25	33.78%	13	20.00%	12	33.33%
M stage	M0	70	94.59%	63	96.92%	36	100.00%
	M1	4	5.41%	2	3.08%	0	0.00%
TNM stage	I	27	36.49%	10	15.38%	13	36.11%
	II	8	10.81%	12	18.46%	22	61.11%
	III	15	20.27%	20	30.77%	1	2.78%
	IV	24	32.43%	23	35.38%	0	0.00%
DUSP11	Low	41	55.41%	30	46.15%	16	44.44%
	High	33	44.59%	35	53.85%	20	55.56%

^#^represents 5 cm for iCCA and 3 cm for pCCA/dCCA.

### Correlation Between Dual-Specificity Phosphatase 11 and Clinicopathological Factors

To screen the potential clinicopathological variables which may be associated with DUSP11 expression, we analyzed the correlation between the clinicopathological variables and DUSP11 with the chi-square test (**Table 2**). In iCCA, DUSP11 expression was significantly associated with the T stage. High expression of DUSP11 was positively correlated with an advanced T stage (*P *= 0.008), indicating that DUSP11 may be an attributor to iCCA infiltration. In pCCA and dCCA, no clinicopathological variables exhibited a significant correlation with DUSP11 expression.

**Table 2:** The correlations between clinicopathological factors and DUSP11.

Clinicopathologic parameters		iCCA			pCCA			dCCA		
		Low	High	P*	Low	High	P*	Low	High	P*
Age (years)	<60	28	17	0.142	21	19	0.783	14	13	0.439
	≥60	13	16		14	11		6	3	
Sex	Male	20	17	0.815	23	23	0.333	10	11	0.320
	Female	21	16		12	7		10	5	
Tumor size^#^ (cm)	<3/5cm	22	12	0.138	19	18	0.643	14	12	0.739
	≥3/5cm	19	21		16	12		6	4	
Differentiation	Well	7	10	0.388	20	11	0.243^$^	10	7	0.753
	Moderately	22	14		11	14		7	5	
	Poorly	12	9		4	5		3	4	
T stage	T1+2	34	18	0.008	18	10	0.142	8	8	0.549
	T3+4	7	15		17	20		12	8	
N stage	N0	28	21	0.674	30	22	0.213	13	11	0.813
	N1	13	12		5	8		7	5	
M stage	M0	39	31	0.824^$^	34	29	0.933^$^	20	16	
	M1	2	2		1	1				
TNM stage	I	17	10	0.184	5	5	0.753	7	6	0.662
	II	6	2		8	4		12	10	
	III	5	10		11	9		1	0	
	IV	13	11		11	12				

*chi-square test, ^#^represents 5 cm for iCCA and 3 cm for pCCA/dCCA, ^$^represents Fisher test.

### Dual-Specificity Phosphatase 11 Was Correlated With Poor Prognosis in Intrahepatic Cholangiocarcinoma

We performed univariate analysis to evaluate the prognostic significance of DUSP11 and other clinicopathological factors in CCA. All clinicopathological factors and DUSP11 expression were enrolled into the univariate analysis. The Kaplan-Meier method was used to plot the OS curves, and the log-rank test was used to analyze the statistical difference between the subgroups (**Table 3**).

**Table 3:** The univariate analysis of DUSP11 and other clinicopathological factors.

Clinicopathologicparameters		iCCA		pCCA		dCCA	
		5-year OS	P*	5-year OS	P*	5-year OS	P*
Age (years)	<60	36.1	0.435	40.6	0.142	43.8	0.600
	≥60	28.3		36.4		54.7	
Sex	Male	31.5	0.288	37.8	0.789	37.8	0.789
	Female	35.3		35.1		35.1	
Tumor size^#^ (cm)	<3/5 cm	44.7	0.007	41.9	0.329	41.9	0.389
	≥3/5 cm	22.8		41.1		32.9	
Differentiation	Well	35.6		49.0		64.3	
	Moderately	31.9	0.586	10.1	0	48.1	0.851
	Poorly	33.3		0		41.5	
T stage	T1 + 2	39.9	0.219	52.9	0.004	34.8	0.647
	T3 + 4	16.7		27.3		43.8	
N stage	N0	42.4	0.010	46.3	0.006	32.3	0.110
	N1	12.4		0		61.1	
M stage	M0	35.7	0.010	39.1	0.012		
	M1	0		0			
TNM stage	I	47.1		75.0		34.4	
	II	58.3	0.049	26.8	0.049	46.6	0.292
	III	22.5		30.8		0	
	IV	13.0		28			
DUSP11	Low	52.6	0.002	47.5	0.354	50.0	0.459
	High	9.4		25.2		45.9	

*represents analysis with log-rank test; ^#^represents 5 cm for iCCA and 3 cm for pCCA/dCCA.

**Figure 2:**
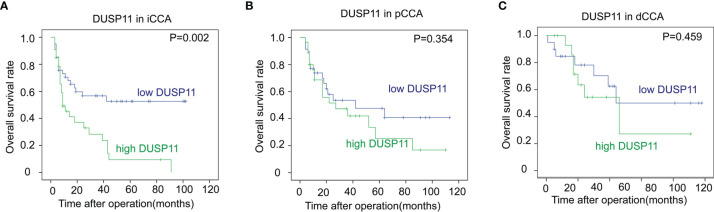
The correlations between DUSP11 expression and the OS rates of CCA. **(A–C)** The survival curves of low and high expressions of DUSP11 in iCCA **(A)**, pCCA **(B)**, and dCCA **(C)** were plotted by the Kaplan-Meier method, and the statistical significance was analyzed with the log-rank test.

In iCCA, a high DUSP11 expression was significantly associated with a low OS rate (*P *= 0.002). The 5-year OS rates of patients with low and high DUSP11 were 52.6% and 9.4%, respectively (**Figure 2A**). However, the prognostic significance of DUSP11 in pCCA and dCCA was not remarkable (*P *= 0.354 and 0.459, respectively), though pCCA and dCCA patients with a high DUSP11 expression seemed to have poorer prognoses compared with those with a low DUSP11 expression (25.2% *vs*. 47.5% in pCCA, 45.9% *vs*. 50.0% in dCCA) (**Figures 2B, C**).

**Figure 3:**
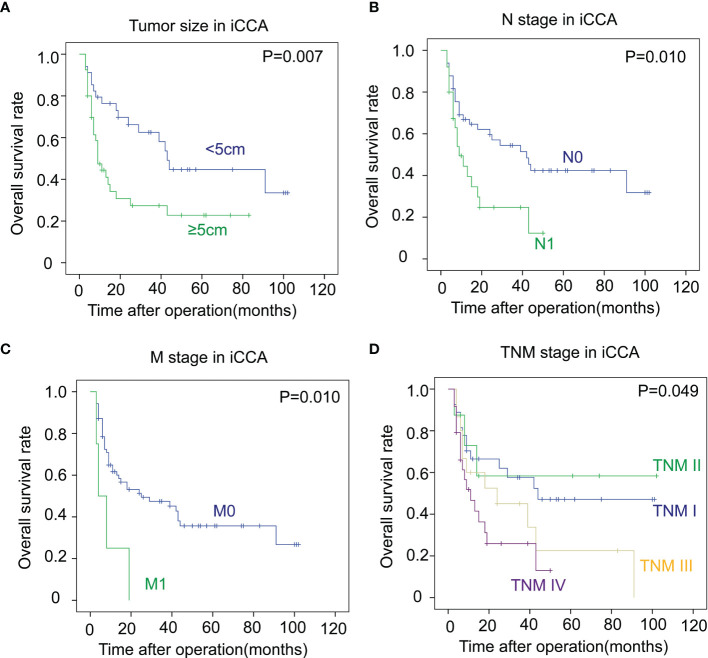
The correlations between the clinicopathological factors and OS rates in iCCA. **(A–D)** In iCCA, the survival curves of tumor size **(A)**, N stage **(B)**, M stage **(C)**, and TNM stage **(D)** were plotted by the Kaplan-Meier method, and the statistical significance was analyzed with the log-rank test.

**Figure 4:**
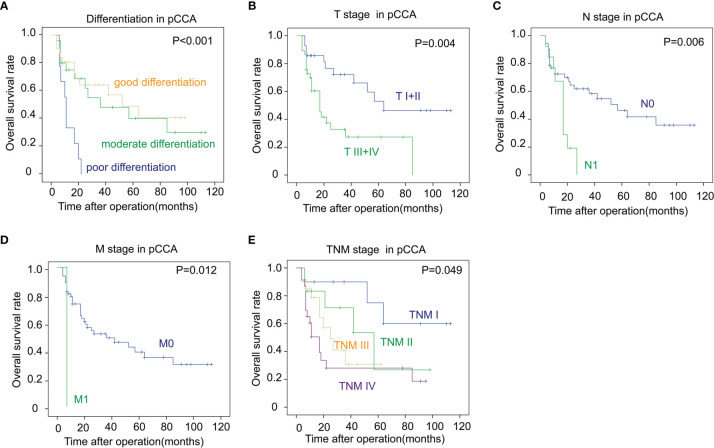
The correlations between the clinicopathological factors and OS rates in pCCA. **(A–E)** Patients with pCCA were stratified according to the tumor differentiation **(A)**, T stage **(B)**, N stage **(C)**, M stage **(D)**, and TNM stage **(E)**, and the correlations between these factors and the OS rates were analyzed with the log-rank test.

In iCCA, a large tumor size and advanced N stage and M stage, representing positive lymphatic invasion and distant metastasis, were also indicators of a poor prognosis (*P *= 0.007, 0.010 and 0.010 respectively) **(** **Figures 3A** **–C**). In addition, an advanced TNM stage was associated with the unfavorable outcome of iCCA as well (*P *= 0.049) (**Figure 3D**). In pCCA, poor differentiation was a notable indicator for a poor prognosis (*P *< 0.001) (**Figure 4A**). Moreover, advanced T stage, N stage, and M stage were also associated with a poor prognosis of pCCA (*P *= 0.004, 0.006, and 0.012, respectively) (**Figures 4B** **–D**). As expected, patients in an advanced TNM stage had a much poorer outcome than those in an early TNM stage (*P *= 0.049) (**Figure 4E**). In dCCA, no factors were defined to be associated with the OS time, which may be attributed to the small number of patients (n = 36).

### Dual-Specificity Phosphatase 11 Was an Independent Prognostic Biomarker of Intrahepatic Cholangiocarcinoma

All the clinicopathological factors were enrolled into the Cox-regression hazard model for multivariate analysis (**Table 4**). In iCCA, DUSP11 was identified as an independent prognostic biomarker (*P *= 0.022, 95% confidence interval = 1.13–4.83). The hazard ratio (HR) of a high DUSP11 was 2.33, representing that patients with a high DUSP11 expression had a 2.33-fold time more than patients with a low DUSP11. However, the independent prognostic significance of DUSP11 in pCCA and dCCA was not significant (*P *= 0.993 and 0.640, respectively).

**Table 4:** Prognostic factors identified by multivariate analysis.

Clinicopathologic parameters		iCCA			pCCA			dCCA		
		HR	95%CI	P*	HR	95%CI	P*	HR	95%CI	P*
Age (years)	<60	1			1			1		
	≥60	1.34	0.69–2.57	0.387	1.98	0.82–4.75	0.129	0.83	0.17–3.93	0.810
Sex	Male	1			1			1		
	Female	0.84	0.45–1.62	0.575	0.85	0.36–1.99	0.702	0.59	0.17–2.10	0.415
Differentiation	Well	1			1			1		
	Moderately+Poorly	0.89	0.40–1.99	0.773	1.98	0.87–4.50	0.103	1.16	0.36–3.77	0.802
Size^#^	<3/5 cm	1			1			1		
	≥3/5 cm	1.76	0.89–3.48	0.107	1.29	0.60–2.74	0.516	0.49	0.09–2.55	0.397
T stage	T1 + T2	1			1			1		
	T3 + T4	0.83	0.40–1.71	0.604	2.15	0.99–4.71	0.057	1.60	0.45–5.72	0.467
N stage	N0	1			1			1		
	N1 + 2	1.66	0.81–3.40	0.167	2.67	1.14–6.23	0.023	0.37	0.06–2.12	0.262
M stage	M0	1			1					
	M1	2.63	0.78–8.78	0.117	5.44	1.02–28.96	0.047			
DUSP11	Low	1			1			1		
	High	2.33	1.13–4.83	0.022	1.01	0.47–2.13	0.993	1.31	0.42–4.17	0.643

*Cox-regression model.

^#^represents 5 cm for iCCA and 3 cm for pCCA/dCCA.

### Prognostic Significance of Dual-Specificity Phosphatase 11 in Extrahepatic Cholangiocarcinoma

**Figure 5:**
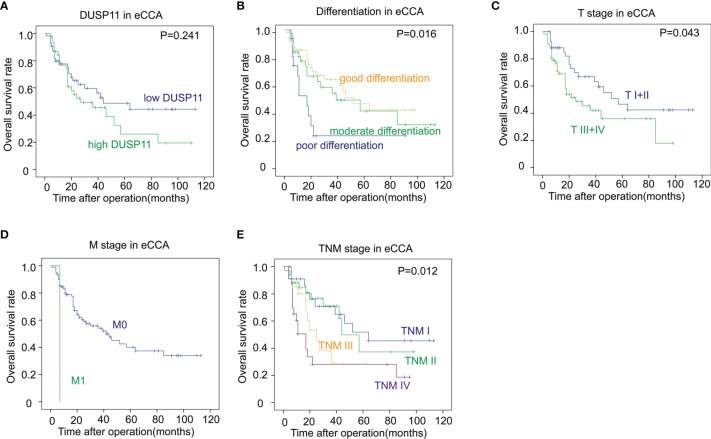
The correlations between DUSP11, clinicopathological factors, and the OS rates in eCCA. **(A–E)** In the 101 cases of eCCA, the associations between the OS rate and DUSP11 expression **(A)** or clinicopathological factors including tumor differentiation **(B)**, T stage **(C)**, M stage **(D)**, and TNM stage **(E)** were analyzed with the log-rank test.

To eliminate the effects of a small sample size towards the statistical significance, we merged pCCA and dCCA to extrahepatic CCA (eCCA), and performed the univariate analysis. In the 101 cases of eCCA, the prognostic significance of DUSP11 was still not remarkable (*P *= 0.241), but there existed a trend that a high DUSP11 expression seemed to correlate a low OS rate (**Table 5**). The 5-year OS rates of low and high DUSP11 were 48.7% and 26.0%, respectively (**Figure 5A**). Moreover, poor differentiation (*P *= 0.016), and advanced T stage (*P *= 0.043), M stage (*P *= 0.003), and TNM stage (*P *= 0.012) were all indicators for an unfavorable prognosis of eCCA (**Figures 5B** **–E**).

**Table 5:** The univariate analysis for eCCA.

Clinicopathologic parameters		eCCA	
		5-year OS	P*
Age (years)	<60	39.6	0.313
	≥60	39.6	
Sex	Male	38.1	0.372
	Female	39.0	
Tumor size^#^ (cm)	<3 cm	39.5	0.886
	≥3 cm	40.4	
Differentiation	Well	45.7	
	Moderately	40.4	0.016
	Poorly	22.1	
T stage	T1 + 2	46.7	0.043
	T3 + 4	36.0	
N stage	N0	41.7	0.484
	N1	29.1	
M stage	M0	40	0.003
	M1	0	
TNM stage	I	51.9	
	II	37.3	0.012
	III	28.6	
	IV	28.0	
DUSP11	Low	48.7	0.241
	High	26.0	

*log-rank test.

^#^represents 5 cm for iCCA and 3 cm for pCCA/dCCA.

## Discussion

Compared with other common cancer types such as gastric cancer or lung cancer, CCA is characterized by its low rate of radical resection because of the specificity of the hepatic portal (19, 20). The low rate of radical surgery increases the difficulty of specimen obtainment and establishment of a large cohort (20, 21), which is the basement of biomarker identification and new drug target. However, more prognostic biomarkers of CCA are an urgent need to select the high-risk patients and help formulate the precise treatment. Our study collected a total of 174 cases of CCAs, which was a relatively large CCA cohort. We demonstrated for the first time that DUSP11 was an independent prognostic biomarker in CCA, suggesting that a high expression of DUSP11 was a post-operational risk and detecting DUSP11 could guide the individual treatment for patients with CCA.

In the seventh edition of AJCC/UICC in 2007, pCCA and dCCA were separated from eCCA and regarded as distinct subtypes (22). iCCA, pCCA, and dCCA have different morbidities, clinical characteristics, treatment strategies, and prognosis, but whether they have different biological features is still controversial (23). In some occasions, iCCA and eCCA have the same biomarkers such as EGFR and HER2 (24, 25), but several biomarkers exhibited a different prognostic significance in iCCA and eCCA (21, 26). In this study, we demonstrated that DUSP11 expression was correlated with a poor prognosis in iCCA but not eCCA. This result further supported that iCCA and eCCA are two distinct cancer types, which have different biological factors and biomarkers.

In the era of high-throughput sequencing, numerous genetic alterations, such as the mutation, deletion, duplication, or translocation of PTP genes, are reported to be linked with diverse cancer phenotypes (27). The underlying mechanisms of the tumor suppressor or oncogenic role of PTPs in tumorigenesis or tumor progression are not fully understood. The loss or genetic alterations of several PTPs are shown to promote tumorigenesis, proliferation, and metastasis in *in vitro* and *in vivo* models, and these PTPs are generally considered to be tumor suppressors, including PTEN in prostate and breast cancer (28–30), SHP1 in leukemia and lymphomas (31, 32), PTPRF in colon, breast, and lung cancer (33, 34), and DUSP4 in breast, pancreas, and thyroid cancer (35–38). On the other hand, several tumor PTPs were identified as tumor suppressors because their genetic variations or loss facilitate tumorigenesis and tumor progression. For example, SHP2 was considered as an oncogene in breast cancer, leukemia, and gliomas (39–43), and PTP4A3 is an oncogene in breast, gastric, and colon cancer (44–46). The studies of DUSP11 in cancer are very few and the role of DUSP11 in tumor progression is nearly in vacancy. This is the first study reporting a definite role of DUSP11 as an oncogene because it is significantly associated with iCCA prognosis. Our results expand the understanding of the role of the DUSP family in cancer, and indicates DUSP11 as a potential drug target of CCA.

Although PTPs are attracting more and more attention as an onco-protein or a tumor suppressor, the improvement of PTP inhibitors as specific inhibitors or target drugs remains challenging. To obtain the specific small-molecule inhibitors are difficult because the catalytic domains of PTPs are very conserved. Till now, only a small proportion of PTPs have specific inhibitors, such as PTP1B, SHP2, and PTPN9 (47–50). However, the interacting proteins, substrates, and molecular catalytic mechanism of DUSP11 are poorly understood, and there is still no available inhibitor of DUSP11. More studies on the expression profile of DUSP11 in cancer would help improve the therapeutic use of its potential inhibitor. We showed the clinical relevance of DUSP11 in CCA and suggested that the inhibitor towards DUSP11 may be a potential therapeutic strategy to CCA.

In conclusion, we, for the first time, investigated the expression of DUSP11 in 174 cases of CCAs, including 74 iCCAs, 64 pCCAs, and 36 dCCAs, and evaluated the clinical significance of DUSP11 by assessing DUSP11 correlation between the clinicopathological factors and prognosis. As a result, we demonstrated that DUSP11 expression was associated with tumor infiltration and the OS rate in iCCA, but not in pCCA or dCCA. DUSP11 was an independent biomarker of iCCA, indicating a poor prognosis. Our results suggested that a high expression of DUSP11 was a post-operational risk factor, and detecting DUSP11 could guide the individual treatment for patients with CCA.

## Data Availability Statement

The raw data supporting the conclusions of this article will be made available by the authors, without undue reservation.

## Ethics Statement

The studies involving human participants were reviewed and approved by the Ethics Committee of Chinese Academy of Medical Sciences and Peking Union Medical College and National Cancer Center Shenzhen Hospital. The patients/participants provided their written informed consent to participate in this study.

## Author Contributions

Concept and design: XC. Administrative support: LX and PW. Specimen collection: XC, LX, PW, WZ, WL, and TL. Collection and assembly of data: XC, LX, PW, WZ, WL, and TL. Data analysis and interpretation: LX and PW. All authors contributed to the article and approved the submitted version.

## Funding

The study is supported by Sanming Project of Medicine in Shenzhen (No. SZSM202011010) and Sanming Project of Medicine in Shenzhen (No. SZSM201911008).

## Conflict of Interest

The authors declare that the research was conducted in the absence of any commercial or financial relationships that could be construed as a potential conflict of interest.

## Publisher’s Note

All claims expressed in this article are solely those of the authors and do not necessarily represent those of their affiliated organizations, or those of the publisher, the editors and the reviewers. Any product that may be evaluated in this article, or claim that may be made by its manufacturer, is not guaranteed or endorsed by the publisher.
